# Body coloration as a dynamic signal during intrasexual communication in a cichlid fish

**DOI:** 10.1186/s40850-021-00075-9

**Published:** 2021-05-01

**Authors:** Leonie John, Ingolf P. Rick, Simon Vitt, Timo Thünken

**Affiliations:** 1 Institute for Evolutionary Biology and Ecology, University of Bonn, An der Immenburg 1, 53121 Bonn, Germany; 2 Institute of Evolution and Ecology, University of Tuebingen, Auf der Morgenstelle 28E, 72076 Tübingen, Germany; 3 Institute of Zoology, University of Bonn, Meckenheimer Allee 169, 53115 Bonn, Germany

**Keywords:** *Pelvicachromis taeniatus*, dynamic signaling, communication, intrasexual aggression, color measurement

## Abstract

**Background:**

Intrasexual competition over access to resources can lead to aggression between individuals. Because overt aggression, i.e. fights, can be costly for contestants, the communication of aggressive motivation prior to engagement in a physical fight is often mediated by conventional signals. Animals of various taxa, including fishes, display visual signals such as body coloration that can dynamically be adjusted depending on the individual’s motivation. Male individuals of the West African cichlid *Pelvicachromis taeniatus* express a yellow body coloration displayed during courtship but also in an intrasexual competition context.

**Results:**

Within-individual variation in male yellow body coloration, as quantified with standardized digital photography and representation in a CIELab color space, was examined in a mating context by exposing males to a female and in a competitive intrasexual context, i.e. in a dyadic contest. Additionally, spectrometric reflectance measurements were taken to obtain color representations in a physiological color space based on spectral sensitivities of our model species. Exposure to females did not significantly affect male color expression. However, analysis of body coloration revealed a change in within-individual color intensity and colored area after interaction with a male competitor. In dominant males, extension of coloration was positively correlated with restrained aggression, i.e. displays, which in turn explained dominance established between the two contestants.

**Conclusion:**

Body coloration in male *P. taeniatus* is a dynamic signal that is used in concert with display behavior in communication during intrasexual competition.

## Background

In territorial species, individuals compete with conspecifics for access to limited resources like territories or mating partners, leading to aggression and fights, which often goes along with establishment of a social hierarchy [1, 2]. Subordinate individuals might try to seek access to resources by challenging dominant individuals, which are in turn aggressively defending their territory. Overt aggression such as physical attacks and fights, however, should be avoided as they are costly, bearing the risk of injury and death for both competitors [3]. Aggressive motivation can be communicated in form of restrained aggression, displaying threat signals towards the competitor [4]. Certain signals can tell a receiver about the sender’s potential to defend its resources and can therefore allow an assessment of the competitor and the risk a fight would hold [5, 6]. Communicating in advance can therefore prevent overt aggression and minimize the risk of a decrease in fitness for both competitors.

Body coloration as a visual signal in social and sexual contexts has been investigated in several invertebrates [e.g. 7–9] and vertebrates [e.g. 10–14], including different fish species [e.g. 15–19]. While morphological traits, e.g. body size are considered honest signals because they are static and reliably give information about an individual’s competitiveness [5, 20], other visual signals are less reliable because they can be easily manipulated and might be inconsistent and deceptive [21, 22]. The reliability of color signals is often ambiguous [3], however in certain cases, they can function as an honest signal [23]. This is especially true for coloration based on carotenoids, whose production is energetically costly as vertebrates cannot synthesize carotenoids themselves, and therefore indicates the physical condition of an individual [24]. Hence, individuals can use display of carotenoid-based coloration to communicate their aggressiveness, threat and fighting potential towards a competitor [3, 25].

A trait like coloration is only informative if it shows a certain amount of variation within the population. For an honest signal, this is synonymous to variation in individual quality [26, 27]. When variation does not only occur between individuals but also within an individual, the trait will often be regarded as inconsistent and deceitful [3]. On the other hand, it holds potential for a high behavioral flexibility [28, 29] and can be used as a dynamic signal [30]. If coloration can be changed rapidly within seconds to hours, it is thought to be relevant for either physiological regulation, concealment or communication in behavioral contexts [31]. It can be adjusted to communicate different information, e.g. signaling dominance or submission towards a competitor [11, 30]. Male three-spined sticklebacks, *Gasterosteus aculeatus,* show an increase in their red nuptial coloration when facing another reproductively active male, indicating the use of this color pattern as a dynamic signal in intrasexual competition [32]. Females of this species change coloration dynamically during their breeding cycle, probably as a signal in reproductive contexts [33]. Different cichlid species have been shown to actively alter their coloration in combination with aggressive or submissive behavior, inducing or avoiding a fight with competitors, respectively [34, 35]. The cichlid *Astatotilapia burtoni* can change between yellow and blue coloration, with yellow males usually dominating competitors [29]. Here, coloration is therefore strongly correlated with dominance in intrasexual competition.

*Pelvicachromis taeniatus* is an African, monogamous cichlid fish with mutual mate choice and bi-parental brood care [36–38]. Individuals of both sexes show an intense, sex-specific nuptial coloration that is relevant during mutual mate choice, as it is related to individual quality [39, 40]. Female body coloration is also involved in female-female competition [40, 41]. In males, the yellow coloration of the ventral side is produced by carotenoids [39]. For reproduction, dominant males are occupying a breeding cave, which they aggressively defend against hetero- and conspecific intruders [42, 43]. Aggression and defense are expressed by biting the opponent, mouth fighting, chasing, and by an offensive display, in which an individual presents its colorful yellow ventral side laterally towards the competitor. Subordinate individuals on the other hand signal their inferiority by becoming dull if attacked or after losing a fight, and by displaying a black dorsal bar. Such displays can occur rapidly within seconds, and are usually associated with submissive behavior, such as hiding and actively avoiding a fight. Previous studies on *P. taeniatus* primarily focused on variation and function of female body coloration in male mate choice and female intrasexual competition [39, 41, 44]. The intensity of female red body coloration increases until spawning; immediately after spawning females become suddenly dull. Extension of blue coloration moreover indicates female fecundity and genetic quality [41], hinting at the importance of body coloration as a dynamic signal in this species. However, the function of male coloration in inter- and especially in intrasexual selection is less well investigated in *P. taeniatus*. While there is experimental evidence that yellow body coloration is indeed connected to male individual quality, female preference for more intensely colored males also depends on their own quality [39].

The aim of the present study was to quantify within-male variation in body coloration in *P. taeniatus* and its use as a signal in communication in different contexts. Within-individual temporal variation and consistency of color expression of males was examined in two different contexts, i.e. mating and intrasexual competition. Furthermore, in the competition context, we examined the interrelationship between male coloration and overt and restrained aggression and dominance. For this purpose, males were presented a competitor in a dyadic contest to observe aggressive and submissive behavior in combination with possibly changing intensity and extension of body coloration.

## Results

Both yellow intensity of male ventral coloration, i.e. Lab-chromaticity (LC) and the relative colored area (RCA) measured before trials did not differ significantly when compared between the two contexts (LC: Wilcoxon rank sum test, W = 740, *n* = 83, *p* = 0.313; RCA: Welch two sample t-test, *t* = -1.285, df = 68.253, *p* = 0.203). Measurements of RCA were repeatable before and after trials for the mating context, but not for the competition context (Table 1). Measurements of LC were not repeatable in both contexts. Coloration measurements of females used in the mating context were repeatable.

**Table 1:** Repeatability of color measurements in male *P. taeniatus* taken before and after trials examined in a competition and mating context and in female *P. taeniatus* used in the mating context

Context	Sex	Variable	***n***	***R***	SE	CI	***p***
Competition	Male	RCA	46	< 0.001	0.083	[0, 0.27]	> 0.999
Competition	Male	LC	46	0.041	0.095	[0, 0.304]	0.420
Mating	Male	RCA	37	0.496	0.141	[0.172, 0.717]	**0.002**
Mating	Male	LC	37	0.264	0.139	[0, 0.522]	0.060
Mating	Female	RCA	36	0.289	0.145	[0, 0.557]	**0.046**
Mating	Female	LC	36	0.409	0.145	[0.074, 0.64]	**0.007**

*R* Repeatability, *SE* standard error, *CI* confidence intervals and *p*-values of *RCA* relative colored area and *LC* Lab-chromaticity. Significant *p*-values (< 0.05) are printed in bold.

**Fig. 1:**
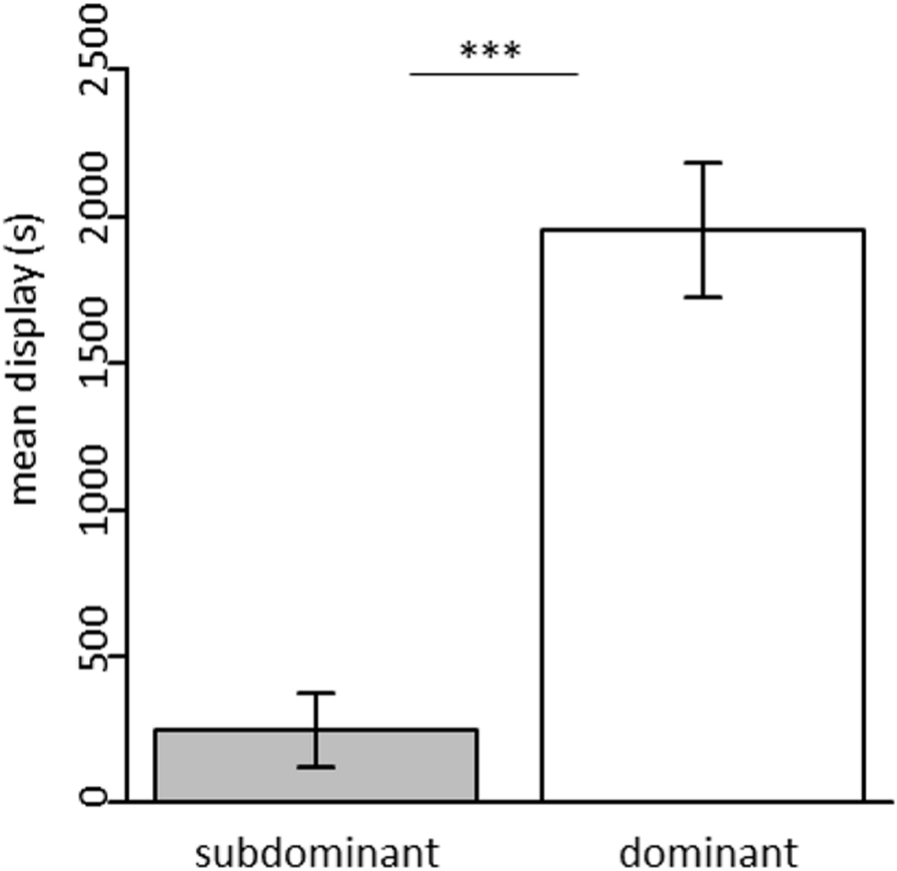
Restrained aggression (displays) and dominance status. Mean display (s) with standard error shown for subordinate (grey) and dominant (white) males. *** *p* < 0.001

**Fig. 2:**
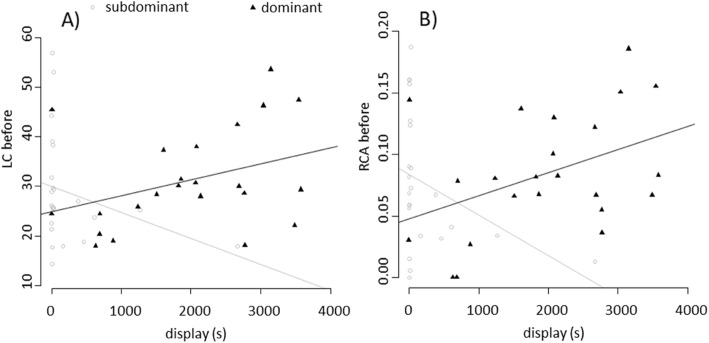
Relationship between restrained aggression, i.e. displays and male coloration. **a** Correlation between Lab-chromaticity (LC) and display and **b** between relative colored area (RCA) and display for dominant (black triangles) and subdominant (grey circles) males with least-squares regression lines

Dominant males showed more displays as a restrained form of aggression than subdominant males, but not more overt aggression, measured in attacks (Table 2, Fig. 1). Size or initial coloration did not predict dominance (Table 2). The relationship between coloration and displays differed between males, depending on dominance status (interaction effect, Table 3, Fig. 2). In dominant individuals, number of displays positively correlated with RCA and LC (Table 4). For subordinate individuals, we found a negative correlation between displays, RCA and LC, respectively (Table 4).

**Table 2:** Summarized results of generalized linear mixed-effects model (glme) for all individuals

Dependent Variable	Explanatory Variable	χ^**2**^	***p***
dominance	display	21.619	**< 0.001**
	attacks	0.056	0.814
	LC before	0.163	0.687
	RCA before	0.025	0.874
	SL	0.502	0.479

Family and trial ID were included as random factor in each model. During stepwise model reduction, degrees of freedom always differed by one. Significant results are printed in bold (*p* < 0.05)

*LC* Lab-chromaticity, *RCA* relative colored area, *SL* standard length

**Table 3:** Summarized results of linear mixed-effects models (lme) for all individuals

Dependent variable	Explanatory variable	χ^**2**^	***p***
RCA before	display x dominance	6.976	**0.008**
LC before	display x dominance	5.010	**0.025**

Family and trial ID were included as random factor in each model. x describes an interaction term. During stepwise model reduction, degrees of freedom always differed by one. Significant results are printed in bold (*p* < 0.05)

*LC* Lab-chromaticity, *RCA* relative colored area

**Table 4:** Summarized results of linear mixed-effects models (lme) for dominant or subordinate individuals only

Dependent variable	Dominance	Explanatory variable	χ^**2**^	***p***
RCA before	dominant	display	4.378	**0.036**
LC before	dominant	display	3.106	0.078
RCA before	subordinate	display	4.770	**0.029**
LC before	subordinate	display	3.145	0.076

Family was included as random factor in each model. During stepwise model reduction, degrees of freedom always differed by one. Significant results are printed in bold (*p* < 0.05)

*LC* Lab-chromaticity, *RCA* relative colored area

## Discussion

Body coloration of male *Pelvicachromis taeniatus* was quantified based on digital photography and additional reflectance spectrometric measurements combined with visual modeling. The latter method was used to get an estimate of the suitability of digital photography for analyses of dynamic color expression in our model species. Spectrometry estimates coloration by measurement of reflected wavelengths, including those not visible for humans, and therefore offers a broad and accurate assessment of body coloration [45, 46]. However, differentiating between components of a trait, such as color intensity and area is necessary to understand the complexity of the trait and its use as a signal [20, 47]. This is more easy to do with photo-variables, as coloration can be measured more easily over an area, while spectrometry offers point-measures only [48]. Evaluation of different color variables showed that both methods of quantification presented in our study produce similar data. The strong correlation of color variables based on spectrometry and calculated in a perception model, and of variables calculated from photographs, indicates that the respective variables are similarly informative. While other studies suggest that an adjustment to a perceiver’s visual system is necessary [49], our results show that in the case of *P. taeniatus*, different variables can be used in combination. Maximum absorbance of opsin types found in *P. taeniatus* are similar to those of humans [50, 51], therefore the use of digital photographs and a color space adjusted to human vision seems reasonable. Moreover, fish in our study were always photographed first, followed by spectrometric measurements. Even though we tried to minimize stress for all individuals, and did not observe severe darkening as a possible stress reaction, handling might still have had an influence on fish coloration and therefore decrease the reliability of spectrometric measurements. Data collected from photographs should therefore be more reliable in our study. Hence, we used photo-based variables in our study to examine the relationship between male aggression and coloration.

Comparison of color measured before and after behavioral experiments suggests that males show a significant temporal within-individual variation which depends on context. Males tested in the competition context in our study could interact freely with a male competitor for 20 hours, while in the mating context they were allowed to interact with a female through a perforated transparent wall for 19.5 hours. As indicated by high repeatability values, within-individual variation in coloration of males and females was less affected by intersexual interactions. In contrast, as indicated by low repeatability values, within-male variation in coloration was affected by male-male competition. In males, coloration seems to be used as a flexible, adjustable signal to communicate threat and aggressive motivation on the one hand or submission on the other hand. This accounts especially for the extension of the colored area, which is contrary to general assumptions that size of a color patch is more static than color intensity [52]. Dynamic change in color intensity is more often considered as an important component of signaling and communication in fishes [18, 53, 54].

In the mating context, coloration remained stable at a high value that is necessary to attract females [39]. In this experiment, males were not able to interact with each other and hence it is not surprising that males did not change their coloration over the trials but rather stayed colorful because in contrast to direct male-male competition no social costs were involved with maintaining the bright ornamentation. It is likely that the males perceived the presence of a different male via olfactory cues which might contribute to continuous high expression of the yellow ornamentation.

Results from the competition context strongly imply the use of body coloration as a dynamic signal in intrasexual communication of aggression for male *P. taeniatus* [30]. Because in *P. taeniatus* yellow coloration is produced by carotenoids [39], it can be an honest, but also dynamic signal and hence is suitable to signal resource holding potential as well as aggressive motivation towards a competitor [5]. Signaling submission on the other hand is associated with darkening, reduction in color patch size and display of a black lateral bar. Those bars are known in fish communication as signals in different contexts, including aggression but also cooperation [55]. Atlantic salmon show a similar signaling and behavior when losing a fight for territory; darkening in color is here correlated with an instant decrease of aggression [54]. Rapid changes in coloration, like sudden dulling or display of dark patterns, are caused by the arrangement of pigments in chromatophores [28]. While intensity of yellow carotenoid-based coloration is considered an honest signal because it reflects resource allocation [56], the cost of reducing the coloration may be negligible and socially mediated (e.g. [57]). By darkening, less competitive males may signal submission, thereby avoiding the social costs that are associated with the expression of conspicuous ornamentation, i.e. being attacked by dominant competitors. In the present study, we did not directly measure the dominant individual’s response to the other individual’s darkening. Future studies should also quantify the response of the observer, as a comprehensive understanding of complex signaling systems requires the examination of the mutual interactions between sender and receiver [3].

While measures of behavior and coloration are often done independently [30], we here studied the interrelationship between male body coloration, aggressive behavior and dominance. Specific or enhanced coloration is frequently correlated to dominance [58, 59], as it can be an indicator of physical condition and resource holding potential [5, 60]. In our study, dominance was explained by the number of displays (restrained aggression) a male exercised, while the number of attacks (overt aggression) did not predict the winner of a contest. Variation in color and body variables between individuals did not explain dominance. Also, the relationship between coloration and aggression was not uniform but dominance-dependent. In dominant males, the color intensity and the extension of the colored area correlated positively with the number of displays; a negative correlation was found in subdominant males. This suggests that the yellow coloration of male *P. taeniatus* contributes to signal a male`s competitiveness. Social experience made by the males before the experiment may also affect dominance in the trials. Accordingly, the establishment and maintenance of dominance in male *P. taeniatus* seems to be determined not by single traits but to be mediated through an interplay of behavior, i.e. displays and color signals.

## Conclusions

Our study shows that photography and image analysis can be an appropriate method to assess coloration in *Pelvicachromis taeniatus*. Male *P. taeniatus* can dynamically adjust their ventral yellow coloration depending on social context. While it is a static signal of quality in a mate choice context, yellow coloration is flexibly altered during intrasexual communication and mediates dominance in concert with display behavior.

## Methods

### Experimental animals

Experimental animals were sexually mature individuals of the species *Pelvicachromis taeniatus*. Size of male individuals varied between 5.0 cm and 7.0 cm (mean 6.15 cm ± 0.42 SD) in standard length. All fish were lab-bred in the third generation of wild-caught individuals from the Moliwe River in Cameroon. Twelve different families of *P. taeniatus* were used. Before the experiment, fish were kept in mixed-sex sibling groups (tank size: 30 x 30 x 50 cm, 45 L) under a 12:12 h light:dark cycle (True-Light, 58 W, 5500 K). All fish were fed *ad libitum* every day with defrosted food consisting of *Artemia* and red, black and white mosquito larvae (ratio 4:4:2:1). To examine context-dependent within-male variation in body coloration, male *P. taeniatus* were tested in behavioral experiments in two different contexts, a male-male competition and a mating context. In the male-male competition context, two males were competing for a territory in a dyadic contest, while in the mating context, two males were presented a female. For coloration measurement, fish were photographed before and after trials. Additionally, spectrometric measurements of coloration were taken after trials. Aggressive behavior was analyzed and related to coloration for the competition context. Measurements and analyses were done without knowledge of individual identity. After the experiments, all individuals were returned to their original housing tanks and used for further behavioral studies.

### Photographs

**Fig. 3:**
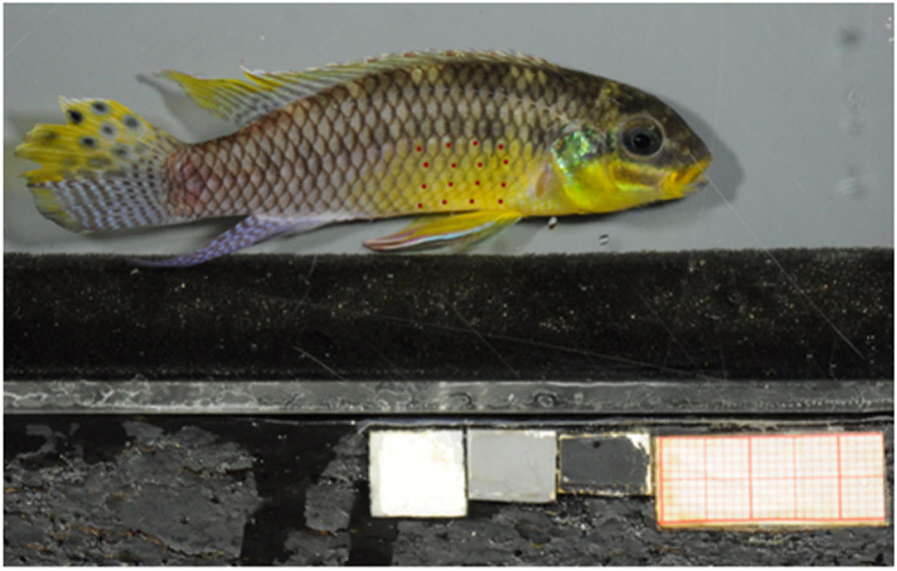
Photograph of a male test individual. Red dots indicate spots for color measurements for the photo analysis

Photographs were taken using a standardized set-up including a photo-box. The photo-box made of Plexiglas (9.5 x 15 x 7 cm, length * width * height, l * w * h) was filled with temperate water (27 °C ± 1 SD) and set up in front of a Nikon D5000 camera with 84 cm distance to the lens (AF-S Micro Nikkor 105mm 1:28G macro objective). Two light sources (16W LED lamps, Toshiba LDRC1665WE7EUD, 32°, 6500K) were used for standardized illumination at a distance of 36 cm and an angle of 35° to the photo-box. A fish was carefully put into the photo-box and held in place by a grey, moveable plastic plate. Photographs were saved in RAW format and color standards (Munsell color standard chip) as well as a size indicator were included on every photograph (Fig. 3). Males show a yellow ornamentation located ventrally that was to be analyzed further. To analyze hue and intensity of coloration, the program Adobe Photoshop CS4 Extended (Version 11.0.2) was used. After brightness was adjusted using the white standard on the photograph and light temperature was adjusted to the light temperature of the light sources (6500K), color samples were taken in form of Lab-values. The color space of the image was set to the CIELab color space [61], which is a three-dimensional space with the L- (lightness), a- and b-value (color) on three axes [62]. Chromaticity in this color space (Lab-chromaticity, LC) can be calculated from a- and b-value: LC = $\sqrt{a^2+{b}^2}$ [63]. For each individual, 16 samples were taken that were evenly spread over the belly (Fig. 3). To determine the extension of the ornamentation, the total lateral body area and the coherent colored area on the ventral side were measured. Total lateral body area was measured manually with ImageJ (Version 1.52a) by selecting the lateral projection area (LPA), excluding fins. Colored area was measured by selecting pixels of a specific color (Table 5) and measuring the area they formed if it was greater than 0.05 cm^2^. Relative colored area (RCA) was then calculated for every fish by dividing colored area by LPA. To test the repeatability of measurements for a single photo, six photos of males and six photos of females were analyzed again for LPA, RCA and LC, approximately eight weeks after the first measurements (Table 6). A high repeatability of these measurements indicated minimal measurement error for this method. To test whether photographs of one individual taken at different times were suitable to produce generally repeatable variables, variables measured in two consecutive trials with the same individuals in the mating context were tested for repeatability (Table 7).

**Table 5:** Color values used to select pixels of colored area for males and females in ImageJ

	Hue	Saturation	Brightness
Males	0 – 64	100 – 255	66 – 255
Females	154 – 255	5 – 255	66 – 255

**Table 6:** Repeatability for color variables measured twice from the same photograph

Variable	***n***	***R***	SE	CI	***p***
LPA	6	0.993	0.016	[0.946, 0.999]	**< 0.001**
RCA	6	1	0.001	[0.997, 1]	**< 0.001**
LC	6	0.988	0.029	[0.917, 0.998]	**< 0.001**

*R* Repeatability, *SE* standard error, *CI* confidence intervals and *p*-values of *RCA* relative colored area and *LC* Lab-chromaticity and *LPA* lateral projection area

Photographs were measured once directly after trials and again several weeks apart. Numbers in bold font indicate significant *p*-values

**Table 7:** Repeatability for color variables of an individual measured after consecutive trials in the mating context

Variable	***n***	***R***	SE	CI	***p***
r_A_	37	0.129	0.131	[0, 0.431]	0.241
RCA	37	0.857	0.053	[0.723, 0.93]	**< 0.001**
LC	37	0.527	0.117	[0.259, 0.714]	**< 0.001**

*R* Repeatability, *SE* standard error, *CI* confidence intervals and *p*-values of *rA* color variables achieved chroma, *RCA* relative colored area and *LC* Lab-chromaticity

Numbers in bold font indicate significant *p*-values

### Spectrometric measurements

**Fig. 4:**
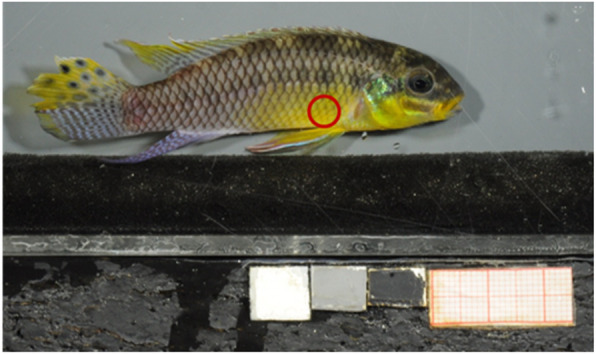
Photograph of a male test individual. Red circle indicates spot for spectrometric measurements

**Fig. 5:**
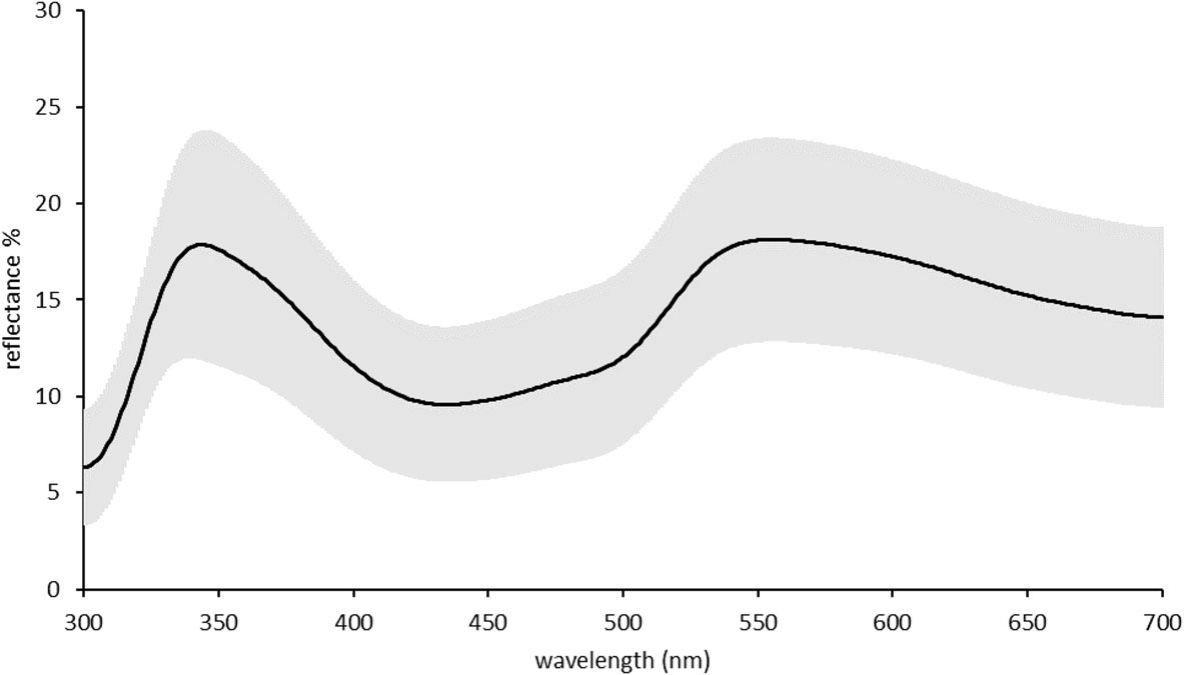
Mean spectral reflectance (black) of yellow body coloration for all males measured in the competition context with standard deviation (grey). Reflectance was measured relative to a white standard

**Fig. 6:**
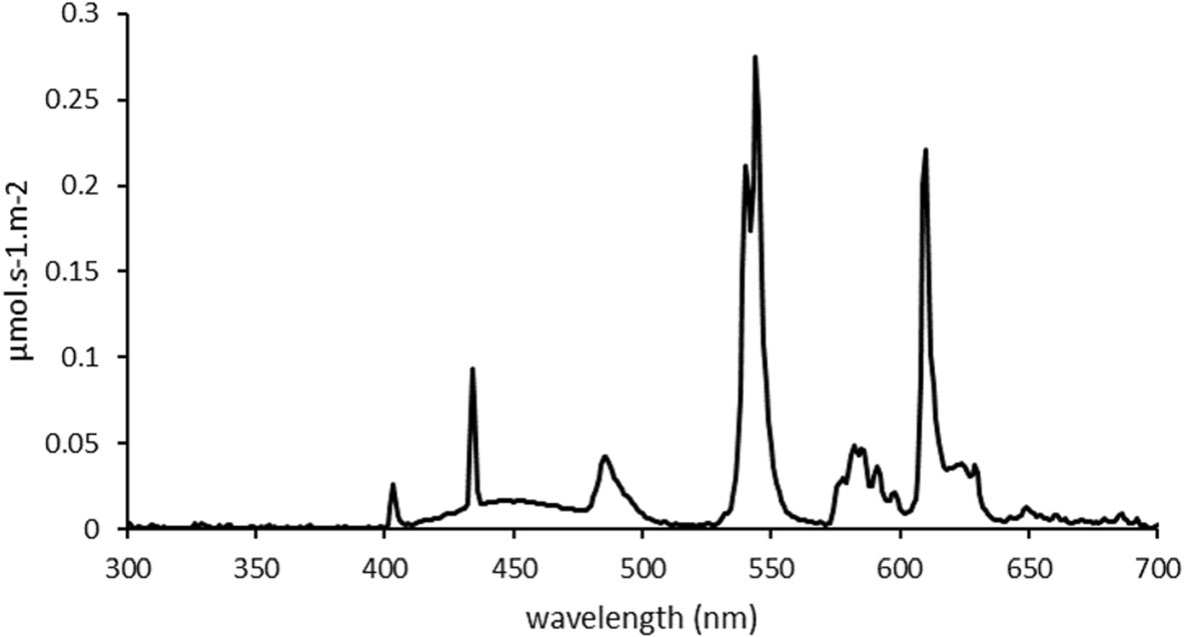
Downwelling irradiance measured in the setup

Even though digital photography is an established method to quantify animal coloration [46, 48], spectrometric measurements of body coloration were done as well, closely following methods described by Vitt et al. [64, 65]. A visual perception model was then created to calculate variables of fish body coloration as viewed by a conspecific, as this might differ from the photo-variables based on human visual properties [66, 67]. The reflectance of the yellow body coloration relative to a 98% (300 – 700 nm) Spectralon white standard (WS-2, Avantes, the Netherlands) was taken with a spectrometer (Avantes Avaspec 2048 spectrometer with AvaLight DHs deuteriumhalogen light source, wavelengths 200 - 1100 nm, program AvaSoft 7.7.2). Only wavelengths between 300 and 700 nm which likely represents the visual range of cichlids [68] were considered for analyses. A test individual was carefully taken out of the water and put on a matt black piece of cloth. The probe of the spectrometer was placed on the colored area (Fig. 4) in a 2 mm distance and at a 45° angle and 10 – 20 spectra were taken per individual and area. This procedure lasted about 20 seconds and was done as quickly as possible to reduce stress for the fish. Spectra were imported into Excel, where a mean spectrum per individual was calculated (from a mean of 11.5 ± 2.18 SD spectra, Fig. 5) and lens transmission of *P. taeniatus* (IPR, unpublished data) was included by multiplication with the spectra. The three main opsin types SWS2A, RH2Aβ and LWS, found in *P. taeniatus* [51] with λ_max_ = 455 nm, 518 nm and 560 nm respectively [69, 70] as well as downwelling irradiance measured in the experimental setup (Fig. 6) were included into a visual perception model. For the opsin types, sensitivity curves were created by using the package “colourvision” in R [71]. Downwelling irradiance in the experimental setup was measured with a portable spectrometer (Avantes AvaSpec 2048 spectrometer connected to a cosine corrector (Avantes CC-UV/VIS), irradiance calibration performed against an Avantes NIST traceable irradiance application standard). Measurements were taken close to the bottom of the tank, in approximately 11 cm water depth from the surface, a depth representing a typical fish position during trials. Because tanks were covered on top with an UV-blocking Plexiglas (GS-233, Röhm) sheet, only light between 400 and 700 nm was present in the setup (Fig. 6). Consequently, the influence of reflectance in the UV spectral range between 300 and 400 nm was negligible in our study. Spectral sensitivity and irradiance were then used on the reflectance spectra in a trichromatic visual model after Endler and Mielke [72] edited by Gomez [73] in the program Avicol (version 5 [73]). In this model, relative excitations of photoreceptors are calculated [72] and used to plot color in a two-dimensional color space [74] which explains chroma (intensity) and hue of the color. Achieved chroma (r_A_) was calculated after Stoddard and Prum [75] as a measure of chromaticity corrected for maximal hue. To test whether spectrometric measurements of one individual taken at different times were suitable to produce generally repeatable variables, variables measured in two consecutive trials with the same individuals in the mating context were tested for repeatability (Table 7). As spectrometric measurements and the visual perception where primarily calculated in addition to the photo-variables and to verify their relevance, variables were compared in a correlation matrix (Table 8). For males, variables from photos (calculated in the CIELab color space) and variables from spectrometry (calculated in a physiological color space) were significantly correlated or tended to be.

**Table 8:** Correlation matrices with body and color variables of males and females

		SL	CI	r_**A**_	RCA	LC
**A)**	**SL**	-	*-0.161*	*0.128*	*0.196*	*0.217*
	**CI**	*0.286*	-	0.101	0.123	0.158
	**r**_**A**_	*0.396*	0.504	-	**0.379**	**0.399**
	**RCA**	*0.193*	0.415	**0.009**	-	**0.906**
	**LC**	*0.148*	0.296	**0.006**	**<0.001**	-
**B)**	**SL**	-	***-0.333***	-0.146	0.032	0.206
	**CI**	***0.044***	***-***	*-0.058*	***-0.396***	***-0.394***
	**r**_**A**_	0.389	*0.734*	-	**0.495**	0.314
	**RCA**	0.858	***0.023***	**0.003**	-	**0.839**
	**LC**	0.222	***0.016***	0.058	**<0.001**	-
**C)**	**SL**	-	**-0.523**	0.171	0.205	0.221
	**CI**	**0.001**	-	-0.025	-0.146	-0.049
	**r**_**A**_	0.320	0.887	-	0.006	0.227
	**RCA**	0.230	0.396	0.970	-	**0.474**
	**LC**	0.195	0.775	0.183	**0.004**	-

Results of correlation tests for A) males in the competition context, B) males in the mating context and C) for females in the mating context. Values in the upper corner represent the correlation coefficient r while values in the lower corner are p-values. Significant p-values are printed in bold (*p* < 0.05). Italic values represent Spearman’s rank correlations while remainder values are based on Pearson’s product-moment correlations

*SL* standard length, *CI* condition index, *r*_*A*_ achieved chroma, *RCA* relative colored area, *LC* Lab-chromaticity

### Behavior

#### Male-male competition context

##### Experimental setup

**Fig. 7:**
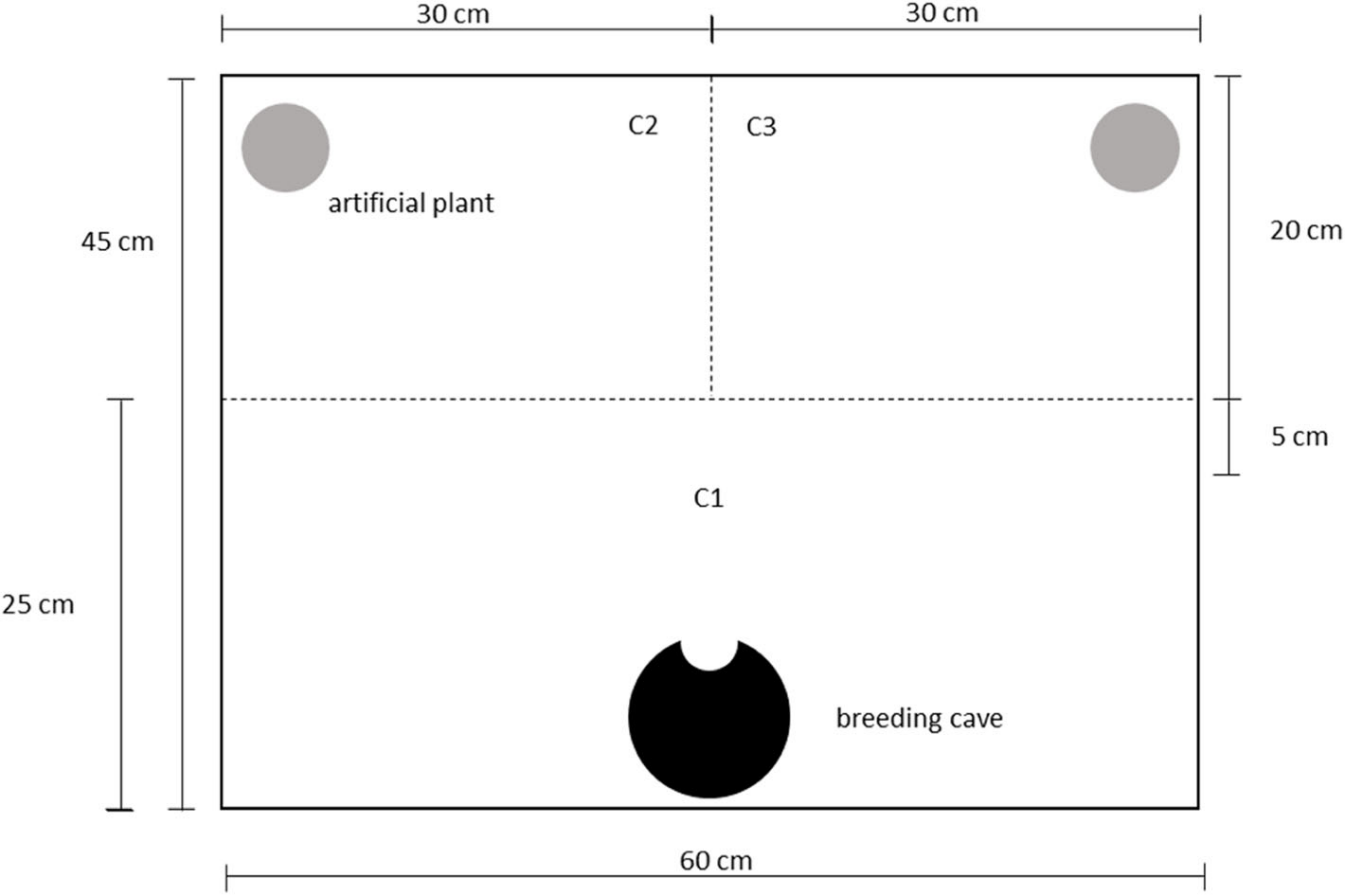
Sketch of the experimental tank from above with dimensions. Before a trial, the tank was divided into three compartments, a big one (C1) with a standardized breeding cave (black circle) and two small ones (C2 and C3) each with an artificial plant (grey circles) for shelter. Inner walls (dashed lines) were removed when a trial started

In order to quantify changes in body coloration in males and relate ornamentation to behavior in a competition context, males were presented a competitor in a dyadic contest. Forty-six males were used for this experiment resulting in 23 pairs and trials, respectively. Males were expected to compete for a potential territory including a breeding cave. An experimental tank measuring 60 x 45 x 30 cm (l * w * h) was first divided into three compartments, a large one measuring 60 x 25 x 30 cm in the back part of the tank and two smaller ones each measuring 30 x 20 x 30 cm in the front of the tank (Fig. 7). The large compartment was separated from both small compartments by a transparent, perforated plate that allowed water flow. The two small compartments were separated by a grey opaque, non-perforated plate to prevent visual contact. The floor was covered with a thin layer of sand. Each small compartment was equipped with an artificial plant to create hiding space. The large compartment was equipped with a standard ceramic breeding cave (bottom Ø 10 cm) positioned centrally at the back wall with the opening directed towards the small compartments, and two air stones for oxygen supply. The back wall as well as the left and the right wall of the tanks were covered with grey opaque walls to prevent any disturbances from the outside. On top, the tank was covered with a transparent Plexiglas plate. Eight experimental tanks were set up simultaneously and filled with half tap water and half substrate-treated water [76] to a level of 13 cm. Water temperature in the tanks was 24.8 °C ± 0.4 SD; water temperature was measured every weekday.

##### Experimental procedure

Two males from different families were selected to create a male pair with a high variability in ornamentation. Intensity and extension of ornamentation was for this purpose first estimated by the experimenter (LJ) to create pairs with a high variation in coloration between contestants. The variation was later confirmed by comparing LC and RCA between the two contestants (Paired t-test; LC: t = 5.075, df = 22, *p* < 0.001; RCA: t = 4.638, df = 22, *p* < 0.001). After capturing the experimental fish from their original tanks, they were first photographed and then their standard length (SL) and mass were measured. Their body condition index (CI) was calculated after Bolger and Connolly [77]. A male pair was then introduced into a tank at 11:00 a.m., with each male being carefully placed into a small compartment. Approximately 26 hours later, the walls separating large and small compartments were removed and both males were able to move freely in the whole tank. Their interaction was recorded by using a webcam (Logitech QuickCam Pro 9000) that was connected to a laptop (Fujitsu Lifebook S Series, Windows 7 Enterprise), conducting four trials at once. Video-recordings were started when the walls were removed and automatically ended after 300 minutes. Another video was recorded for each trial in the morning starting at 7:30, to check for consistency of dominance over the trial. Videos were started remotely without entering the room and were ended after 90 minutes. Males were then removed from the tank and photographed again, directly afterwards the spectrometric measurements were taken (see below). Other behaviors were analyzed from the videos. After experiments, identification of individuals was possible by individual phenotypical traits (e.g. individual spot patterns on fins). Males were fed with the regular food about two hours before the plates in the experimental tank were removed. Every individual was only used once in this experiment.

##### Behavioral analysis

**Fig. 8:**
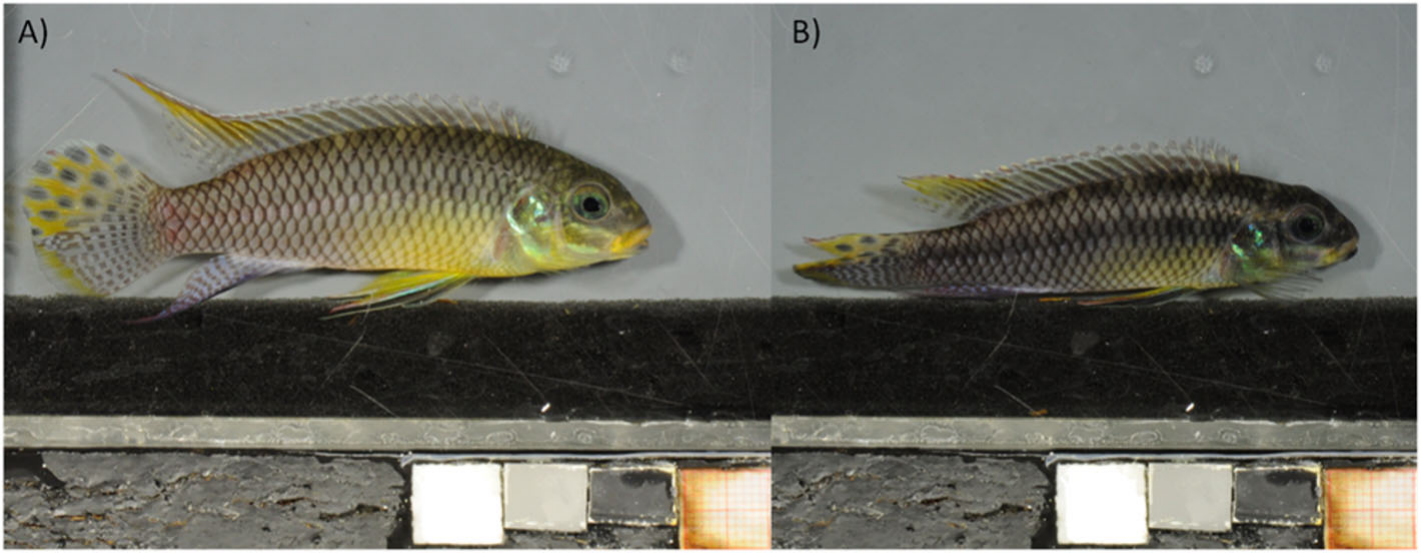
Photographs of males with **a** typical dominant coloration and **b** typical subdominant stress coloration

The behavioral observation software BORIS [78] was used to analyze the videos taken of competition behavior. Observations started when the fish first interacted and was then continued for 60 minutes. Fighting and dominant / sub-dominant behaviors were recorded, such as attacks, mouth fight, chasing and hiding. To clearly determine dominance in a male pair, both individuals had to show clear dominant / subordinate behavior. Crucial was however the subordinate male which had to show stress coloration (Fig. 8) and subordinate behavior (hiding or escaping). In all 23 cases, stress coloration was apparent at least at one point during the observation, therefore it was always possible to determine one subordinate and the according dominant individual. In 21 of these cases this was happening within the 60 minutes observation, while in two cases, the observations had to be extended to determine the dominant male. The observation was then continued for 9 minutes and 95 minutes respectively, not recording behaviors but only the duration until a dominant male could be determined. It was not measured how quick a change in coloration appeared. However, the typical appearance of a dark lateral bar happened rapidly and within a few seconds in all cases. If no individual entered the cave during the 60 minutes, the remaining time of the videos was checked for cave occupation. Videos recorded in the morning were used to check whether dominance of the males were consistent overnight. In all cases, the same male was still dominant in the morning. To assure that none of the experimental fish would suffer from injuries, fish were observed over the remote-control program when starting the trial to possibly interrupt a fight. In no case, fish had to be interrupted. Fish were then left unmonitored overnight. No fish were found injured after the trial.

#### Mating context

##### Experimental setup

In a second experiment, we examined changes in male coloration in a mating context using the same stock of experimental fish. Data of 37 individuals was taken here as additional measures of male body and color variables in a different context. The same experimental setup was used in the mate choice experiment, but the internal walls were never removed. Each small compartment had a breeding cave, the large compartment had two small flowerpots as a hiding place.

##### Experimental procedure

For the mating context experiment, males were captured from their original tanks, size and mass were measured and photographs taken like described above. Both males were introduced into a small compartment of the tank and then were able to acclimate to their compartment for three days. Then, a female was introduced into the large compartment and was able to interact with both males through the transparent, perforated wall for 19.5 hours. Contact between male and female was possible visually and olfactory, between the males only olfactory. With the end of a trial, males were removed from the experimental tank to take photographs and spectrometric measurements. We here do not consider any behavioral variables taken in the mate choice experiment but only use color and body variables measured in this context. Measurements of coloration were taken in the same way as described above for the competition experiment, however time between measurement of variables before and after trials was only two days in the competition experiment while it was four days in the mate choice experiment. Additionally, in the mating context, males were tested a second time with a different female. Those trials are not considered here for analysis of behavioral data, but coloration measurements after the second trials were used for comparison with those from the first trial to check for repeatability of measurements for each individual in a similar situation. Females used in the mating context were also measured for body and color variables to quantify their purple nuptial body coloration. Measurements were taken as described for males, but adjusted to purple instead of yellow body coloration (Table 5). Data collected from females however was not used for further analysis here and is only presented as a comparison to male body coloration variables.

Differences in body and color variables between the two contestants were not significantly different between the two contexts (Wilcoxon rank sum exact test: SL: W = 120, *p* = 0.202; CI: W = 172, *p* = 0.746; LC: W = 149, *p* = 0.722; Welch Two Sample t-test: Mass: t = -0.756, df = 26.96, *p*-value = 0.456; RCA: t = 0.207, df = 20.049, *p*-value = 0.838, Table 9).

**Table 9:** Mean values and mean difference for body and color variables between males.

Variable	Mean ± SD	Mean Difference ± SD
**A. Competition context**		
SL (cm)	6.194 ± 0.379	0.404 **±** 0.282
Mass (g)	5.728 ± 1.061	1.359 **±** 0.891
CI (g/cm^3^)	2.386 ± 0.212	0.227 **±** 0.16
LC	29.946 ± 10.526	13.446 **±** 10.634
RCA (mm^2^)	0.08 ± 0.052	0.071 **±** 0.048
**B. Mating context**		
SL (cm)	6.092 **±** 0.474	0.643 **±** 0.533
Mass (g)	5.3 **±** 1.044	1.592 **±** 0.918
CI (g/cm^3^)	2.331 **±** 0.253	0.24 **±** 0.27
LC	31.969 **±** 10.904	14.99 **±** 12.366
RCA (mm^2^)	0.095 **±** 0.058	0.067 **±** 0.05

Mean and standard deviation for body and color variables for all males, as well as mean difference of these variables between the males of a pair for A) the competition context and B) the mating context, measured before the experiments

*SL* standard length, *CI* condition index, *rA* achieved chroma, *RCA* relative colored area, *LC* Lab-chromaticity

### Statistical analysis

Analysis of the experiment with a competition context was based on 46 males. Thirty-seven of these individuals were also tested in the mating context. For the variable RCA (relative colored area), in the mating context, some data points had to be excluded because the pectoral fin covered coloration on the photographs, leading to 35 measures of RCA after and 33 measures of RCA before. Data was analyzed statistically with the program R (version 3.5.3, [79]). Figures were created using the package ‘GrapheR’ [80]. All data was first tested for normal distribution using the Kolmogorov-Smirnov test with Lilliefors correction. The variable Lab-chromaticity measured before the trials in the competition context was successfully transformed towards normality with a Box-Cox power transformation [81]. RCA and LC measured before trials were compared between the two contexts with a Welch Two Sample t-test and a Wilcoxon rank sum test respectively, to ensure that starting conditions of the males were similar in both contexts.

Repeatability of RCA and LC before and after trials was calculated to check for variation of coloration within an individual over a trial. Tests for repeatability were conducted with a repeatability estimation using the ‘lmm’ function from the ‘rptR’ package in R [82], where 95% confidence intervals were computed with bootstrapping.

For the nominal variable dominance, the effect of size, aggression and color was tested in a generalized linear mixed-effects model (glme) assuming binomial distribution, using the ‘glmer’ function included in the package ‘lme4’ [83]. Random factors were family and trial ID, explanatory variables were display, attacks, size and LC and RCA measured before the trials. The color variables RCA and LC measured before the trials were tested as dependent variables in linear mixed-effects models (lme), using the ‘lmer’ function included in the package ‘lme4’ [83]. Explanatory variables were dominance and display, random factors were family and trial ID. The interaction of display and dominance was calculated with an ANOVA. Data was then separated for dominant and subordinate individuals. With both data sets, the effect of display on the color variables RCA and LC measured before the trials was tested in linear mixed-effects models with the color variable as dependent variable and display as explanatory variable. Random factor was family. In all models, tests of significance of an explanatory variable were based on likelihood ratio test.

## Data Availability

The datasets generated and/or analyzed during the current study are available from the corresponding author on reasonable request.

## Abbreviations

RCA: Relative colored area
LC: Lab-chromaticity
r_A_: Achieved chroma
SL: Standard length
LPA: Lateral protection area
CI: Condition index
